# Seventh Annual Announcement of the Ohio Col. of Dental Surgery

**Published:** 1851-10

**Authors:** 


*Seventh Annual Announcement of the Ohio College of Dental Surgery. Session of* 1851-2.

We are gratified to learn from the above announcement, that the college building and museum, have been purchased by an association of dentists, thus relieving the faculty from the embarrassments under which it had previously labored. The departments under the reorganization are, Principles and Practice of Dental Surgery; Anatomy and Physiology; Pathology and Therapeutics: Operative and Mechanical Dentistry and Chemistry.

We also perceive that the faculty have adopted the plan pursued by the Baltimore College, of appointing an examining committee, to be present at, and to participate in the examination of the candidates for the degree of Doctor of Dental Surgery. This is as it should be.

Most sincerely do we hope that those who are connected with the above institution may be abundantly rewarded for their arduous labors.

